# Editorials, Reviews, Etc.

**Published:** 1872-01

**Authors:** 


### PART III.

### Editorials, Correspondence and Miscellaneous.

### OUR THANKS.

We sincerely thank those gentlemen who have sent us communications. We will publish as early as possible. We trust our friends everywhere will send us articles.

### DELAYED.

The Companion, owing to unavoidable circumstances, has not appeared on the 15th of each month. The present number has been delayed by the pressure on our advertising columns and the Chrismas holdidays. In future our subscribers may expect The Companion between the 15th and 20th of each month.

### FOREIGN CORRESPONDENCE.

We call attention to the interesting letter of Dr. A. W. Calhoun, from Berlin. We have on hand and will publish, in our next, a second letter from this gentleman. We trust he will, at an early day, give our readers the treatment for granular opthalmia, as promised.

### LEFT OUT.

Our miscellaneous correspondence, including the letters of Drs. Taylor and Tucker, were unavoidably crowed out in this number.

